# Comparison of Diagnostic Yield and Safety of Serial Pancreatic Juice Aspiration Cytologic Examination (SPACE) with Different Indications

**DOI:** 10.3390/diagnostics13081498

**Published:** 2023-04-21

**Authors:** Tatsunori Satoh, Shinya Kawaguchi, Shodai Takeda, Yuya Ishiguro, Kazuhisa Asahara, Shuzo Terada, Shinya Endo, Naofumi Shirane, Hideyuki Kanemoto, Kazuya Ohno

**Affiliations:** 1 Department of Gastroenterology, Shizuoka General Hospital, Shizuoka 420-8527, Japanshinya-endo@i.shizuoka-pho.jp (S.E.);; 2 Department of Surgery, Shizuoka General Hospital, Shizuoka 420-8527, Japan

**Keywords:** serial pancreatic-juice aspiration cytologic examination, pancreatic cancer, carcinoma in situ, intraductal papillary mucinous neoplasms

## Abstract

We assessed whether there are differences in the diagnostic yield and safety of serial pancreatic juice aspiration cytologic examination (SPACE) among different indications. We retrospectively analyzed 226 patients who underwent SPACE. They were classified into group A (patients with pancreatic masses, including advanced adenocarcinoma, sclerosing pancreatitis, or autoimmune pancreatitis), group B (suspicious pancreatic carcinoma patients without obvious pancreatic masses, including small pancreatic carcinoma, carcinoma in situ, or benign pancreatic duct stenosis), and group C (intraductal papillary mucinous neoplasm, IPMN). There were 41, 66, and 119 patients, with malignancy diagnosed in 29, 14, and 22 patients, in groups A, B, and C, respectively. The sensitivity, specificity, positive predictive value, negative predictive value, and accuracy were 69%, 100%, 100%, 57%, and 78% in group A; 79%, 98%, 92%, 94%, and 94% in group B; and 27%, 87%, 32%, 84%, and 76% in group C, respectively. PEP was observed in three (7.3%), three (4.5%), and fifteen (13%) patients in group A, B, and C, respectively (*p* = 0.20). SPACE is useful and safe in patients with suspicious small pancreatic carcinoma. However, it has limited efficacy and might not be recommended in patients with IPMN because of the high frequency of PEP.

## 1. Introduction

Pancreatic malignant tumors, especially pancreatic ductal adenocarcinoma (PDAC), are known to have a poor prognosis, with the overall 5-year survival rate reported as only 8.5% in Japan [1]. However, when the tumor is 10 mm or less, the 5-year survival rate is more than 80% [2]. Although, the cancer registry has reported that UICC stage 0 and IA patients accounted for only 1.7% and 4.1% of all PDAC, respectively [2]. To improve the prognosis of PDAC, early diagnosis is essential. For the early detection of pancreatic malignancies, the utility of several markers has been reported, such as CA19-9/CEA level, microRNAs, and other markers [3]. However, making a definitive diagnosis using these markers is difficult, and there is a need for pathological diagnosis. Serial pancreatic juice aspiration cytologic examination (SPACE) under endoscopic retrograde cholangiopancreatography (ERCP) is useful for assessing pancreatic neoplastic lesions [4,5,6,7,8]. For patients with pancreatic mass lesions, endoscopic ultrasonography-guided fine needle aspiration (EUS-FNA) is useful for making a pathological diagnosis with high diagnostic accuracy and low adverse event rate [7,8,9,10,11]. However, EUS-FNA is difficult to perform in cases of carcinoma in situ and when the tumor is small, unclear, or located in an inaccessible lesion. Furthermore, the diagnostic yield of EUS-FNA for small solid pancreatic lesions is insufficient [12]. In contrast, a previous study described the efficacy of SPACE for pancreatic carcinoma (PC) as well as intraductal papillary mucinous neoplasms (IPMN) [13]. Therefore, SPACE has been recognized as an effective method for diagnosing pancreatic neoplasms. A concern for SPACE is post-ERCP pancreatitis (PEP). Several reports described that naso-pancreatic drainage (NPD) placement is a risk factor for PEP [5,13]; however, the risk factors for PEP in patients who underwent SPACE are unclear. Moreover, no comparative studies have examined differences in efficacy and safety for different indications. Therefore, this retrospective study aimed to assess the diagnostic yield and safety of SPACE in patients with any pancreatic neoplasms.

## 2. Materials and Methods

### 2.1. Study Design

We performed a retrospective analysis of consecutive patients who underwent SPACE for the diagnosis of pancreatic neoplasms between January 2013 and August 2021 at Shizuoka General Hospital. Patients who underwent prior pancreatic surgery and those with failed NPD placement were excluded. The final analysis was conducted using follow-up data collected at the end of August 2022. The relevant institutional review board approved this retrospective study (approval number: SGHIRB#2021040), which was conducted in accordance with the principles of the Declaration of Helsinki, and the need for informed consent was waived because of the retrospective nature of the study.

SPACE was performed for pancreatic neoplasms in our institution because of: (1) the presence of a pancreatic mass with difficulty performing EUS-FNA (to diagnose PC); (2) a negative histological diagnosis preceding EUS-FNA in patients with suspected pancreatic cancer (to diagnose PC); (3) the existence of indirect findings of pancreatic ductal adenocarcinoma (PDAC) without obvious pancreatic mass, such as main pancreatic duct (MPD) dilatation/stenosis or a retention cyst without pancreatic mass (to diagnose early stage or in situ PC); and (4) IPMN with worrisome features (WF), high-risk stigmata (HRS), or any risk of high-grade dysplasia (HGD)/invasive cancer (to predict malignant IPMN). Patients who underwent SPACE for reasons (1) or (2) were included in group A, those who went for reason (3) were in group B, and patients who underwent SPACE to predict malignant IPMN were included in group C.

### 2.2. Placement of Endoscopic NPD Tube and SPACE

ERCP was performed using conventional lateral viewing scopes (JF-260V, TJF-260, and TJF-290V; Olympus Medical Systems, Tokyo, Japan). Pancreatography was performed using a standard catheter (ERCP catheter; MTW Co., Ltd., Dusseldorf, Germany). Afterward, the catheter and guidewire were advanced deep into the MPD near the target lesion, and NPD (5Fr/6Fr, QuickPlaceV; Olympus, Tokyo, Japan) was inserted and placed. The endoscopic sphincterotomy was not performed for placing NPD in principle. Finally, SPACE was performed by placing an NPD tube to collect pancreatic juice for two days.

### 2.3. Definitions

Patient information was collected from the medical records and the endoscopic database of our hospital. The MPD diameter was evaluated using the most recent computed tomography or magnetic resonance cholangiopancreatography scan before NPD placement. In principle, the tumor size was measured using EUS-FNA.

Main duct IPMN (MD-IPMN) was defined as the segmental or diffuse dilation of MPD >5 mm without other causes of obstruction, branch duct IPMN (BD-IPMN) was pancreatic cysts >5 mm in diameter that communicate with the MPD, and mixed type IPMN (MX-IPMN) met the criteria for both MD-IPMN and BD-IPMN [14]. In addition, HS or WF were defined according to the revised definitions of the 2017 International Consensus Fukuoka guidelines [14].

Pancreatic juice cytology was performed by a cytotechnologist and a pathologist. The cytological diagnoses were categorized into the following five groups: benign/reactive process (classes 1, 2), atypical cells (class 3a), severe atypical cells (class 3b), strongly suspected malignancy (class 4), and conclusive cytology for malignancy (class 5). At least one cytology was diagnosed as class 3b–5, and SPACE was considered cancer-positive.

The final diagnosis of malignancy was comprehensively assessed based on the surgical pathological findings or clinical course over 12 months after the examination. Resected pancreatic specimens were classified based on histopathological findings. In patients suspected of PC, high-grade PanIN (PanIN-3) and invasive PC were considered malignant. In patients with IPMNs, high-grade dysplasia and invasive carcinoma were defined as malignant. Non-resected suspicious PC lesions and IPMNs were followed up by imaging for at least one year. The lack of progression was regarded as non-malignant within one year. Progression by imaging was defined as follows: (1) diagnosed as pancreatic cancer using EUS-FNA and an increase in the size of the pancreatic mass >20% within one year or the appearance of metastatic disease in patients with a pancreatic mass suspicious of pancreatic cancer; (2) the appearance of a pancreatic mass within one year in patients with suspected pancreatic cancer without a pancreatic mass; (3) increased MPD or mural nodule size of >20% within one year in patients with IPMN with HRS; or (4) the appearance of HRS or the occurrence of mural nodules within one year in patients with IPMN without HRS.

PEP was defined as progressive pain accompanied by an increased serum pancreatic amylase level up to three times the upper normal limit within 24 h after ERCP. The severity of PEP was defined using Cotton’s criteria [15]. The lexicon for endoscopic adverse events (AEs), advocated by the American Society of Gastrointestinal Endoscopy, was used to diagnose and grade the severity of other AEs [16].

### 2.4. Statistical Analyses

Non-parametric values are presented as medians and interquartile ranges and were analyzed using the Kruskal–Wallis test. Categorical variables are presented as proportions and were analyzed using Fisher’s exact test. Univariate analyses were performed to analyze the risk factors for PEP in all patients. The candidate factors were age, sex, MPD size, indication for SPACE, history of acute pancreatitis, location of the target lesion, and size of the NPD tube. All statistical tests were two-tailed and were assessed at a 0.05 probability level. All analyses were performed using R version 3.4.1 (The R Foundation for Statistical Computing, Vienna, Austria).

## 3. Results

### 3.1. Patient Characteristics

**Figure 1:**
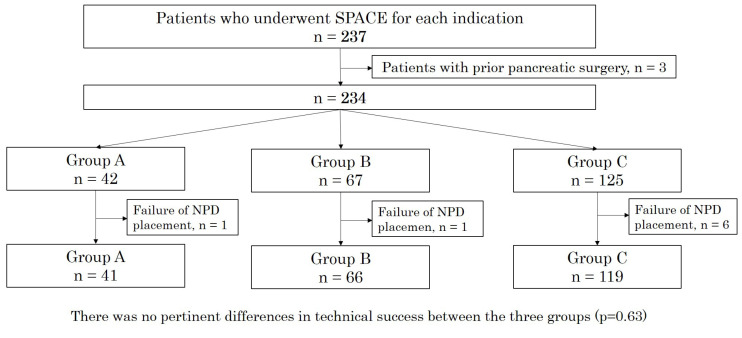
Flow diagram of the study. There were no significant differences in technical success among the three groups (*p* = 0.63). Group A included patients with pancreatic masses, Group B included patients without obvious pancreatic masses, and Group C included IPMN patients. IPMN, intraductal papillary mucinous neoplasm; SPACE, serial pancreatic-juice aspiration cytologic examination; NPD, naso-pancreatic drainage.

During the study period, 237 patients underwent the ERP placement of an NPD tube. Of these, three patients with a history of prior pancreatic surgery and eight patients in whom NPD placement was difficult (group A, one patient; group B, one patient; group C, six patients) were excluded. The remaining 226 patients were included in the analyses and were classified into group A (*n* = 41), group B (*n* = 66), and group C (*n* = 119) (Figure 1). The technical success rate of NPD placement was 98% in group A, 99% in group B, and 95% in group C. There were no significant differences in technical success among the groups (*p* = 0.63). In group C, 12 patients had MD-IPMN, 62 had BD-IPMN, and 45 had MX-IPMN on imaging findings.

Patient characteristics are shown in Table 1. The baseline patient characteristics of the three groups, including age and sex, were similar. Group B included a greater number of patients with a history of acute pancreatitis. MPD size was larger in group C than in group B. In groups A and C, the target lesion was located more frequently in the pancreatic head compared to group B.

### 3.2. Diagnostic Ability of SPACE

The median number of cytology samples was six in each group. Malignancy was observed in 29 patients in group A, 14 in group B, and 22 in group C. The overall sensitivity, specificity, positive predictive value, negative predictive value, and accuracy of SPACE were 79%, 98%, 92%, 94%, and 94% in group A, 69%, 100%, 100%, 57%, and 78% in group B, and 27%, 87%, 32%, 84%, and 76% in group C, respectively (Table 2). In group A, the accuracy of SPACE showed an association with the tumor size based on Fisher’s exact test (*p* < 0.001) (Table 3).

### 3.3. NPD Tube Related Events and ERCP Related Adverse Events

Regarding NPD-related events, none were observed in group A; however, one (1.5%) and five (4.2%) were observed in groups B and C, respectively (*p* = 0.22) (Table 4). PEP was observed in three (7.3%), three (4.5%), and fifteen (13%) patients in group A, B, and C, respectively (*p* = 0.20) (Table 3). All patients who developed PEP underwent repeat cytology, and the NPD tube was withdrawn after the scheduled cytology. PEP was resolved promptly with conservative treatment in all cases. Severe pancreatitis did not occur in any of the groups. The univariate analyses of the risk factors for PEP are shown in Table 5. The rate of PEP tended to be higher in the IPMN patients, without being statistically significant (odds ratio, 2.41; 95% confidence interval, 0.85-7.92; *p* = 0.11).

## 4. Discussion

This study is the first to examine differences in the efficacy and safety of SPACE for each diagnostic indication. SPACE is more effective for the diagnosis of early-stage PC than for the diagnosis of malignant IPMNs, and an IPMN detected by SPACE is a risk factor for PEP.

Pancreatic juice cytology is an important diagnostic modality for pancreatic ductal carcinomas. However, its sensitivity for pancreatic cancer, which ranges from 33 to 76%, is insufficient [17,18,19,20,21]. Recently, it has been reported that pancreatic juice cytology after placement of an NPD, known as SPACE, was effective for the diagnosis of PC [4,5,6,7,8]. Mikata et al. reported that the sensitivity, specificity, positive predictive value, negative predictive value, and accuracy of SPACE for pancreatic cancer are 80%, 100%, 100%, 71%, and 87%, respectively [5]. Furthermore, Iiboshi et al. reported the usefulness of SPACE for carcinoma in situ, with a sensitivity, specificity, and accuracy of 100%, 83.3%, and 95%, respectively [6]. However, the report by Iiboshi et al. included IPMN and a small number of pancreatic cancer patients (the number of PDAC patients was ten). Moreover, the study by Mikata et al. did not examine the difference of SPACE depending on the presence or absence of pancreatic masses and did not compare the effectiveness of SPACE with IPMN. However, our study has the strength of having many patients and a comparative analysis for each indication. Furthermore, it demonstrated a high diagnostic accuracy rate of 94% using SPACE for patients with suspected PC without obvious mass lesions.

Some promising biomarkers for PC detection have been identified, and serum tumor markers, including CA19-9, are known as useful biomarkers for the diagnosis of PDAC [3]; however, the positive rate of tumor markers in patients with stage 0 or IA is not high. According to a study by Ikemoto et al., the levels of carcinoembryonic antigen, carbohydrate antigen 19-9, duke pancreatic monoclonal antigen type 2, and s-pancreas-1 antigen were elevated in 6.9%, 27%, 17%, and 19% of early-stage PDAC patients, respectively [22]. Recently, the utility of several markers has been reported [3]. However, there are no solid results or a consensus on which circulating biomarkers can and should be used in clinical practice [3,23]. Furthermore, there are few satisfactory clinical data of these biomarkers in patients with early-stage pancreatic cancer. Therefore, at present, SPACE, which has high diagnostic accuracy, is considered a useful method for the diagnosis of pancreatic cancer, especially in patients with early-stage pancreatic cancer.

IPMNs can have various histopathologic degrees ranging from adenoma to invasive carcinoma [24], and the exact cytologic discrimination of benign from malignant tumors based on the cytologic findings of pancreatic juice is sometimes difficult [24]. Therefore, the utility of pancreatic juice cytology for the diagnosis of malignant IPMNs remains controversial [25,26,27,28]. There are limited data on the diagnostic ability of SPACE for malignant IPMN, and whether SPACE improves the diagnostic accuracy for malignant IPMNs remains unknown. Furthermore, Yamakawa et al. reported that the sensitivity, specificity, positive predictive value, negative predictive value, and accuracy of SPACE for malignant IPMN were 33.3%, 100%, 100%, 20%, and 42.9%, respectively [13]; however, the study had a small sample size. In contrast, our study demonstrated that the sensitivity, specificity, positive predictive value, negative predictive value, and accuracy of SPACE for malignant IPMN were 27%, 87%, 32%, 84%, and 76%, respectively. There was a gap in the positive and negative predictive value ratios between the two studies. The differences in the number of included patients (SPACE was performed in seven patients in the previous study, and our study included a relatively large number of SPACE cases) was one reason, and the difficulty of diagnosing malignant IPMN based on the cytologic findings might also have contributed to the different results. In addition, the low sensitivity ratio was similar in both studies. In IPMN, if we collect a sufficient volume of pancreatic juice, it might be difficult to identify a few malignant cells among a vast majority of benign cells derived from low-grade dysplasia in the specimen. Uehara et al. described that higher sensitivities of pancreatic juice cytology were yielded only by acquiring specimens directly from the neoplasm in the MPD, whereas lower sensitivities were produced because malignant cells were not aspirated directly, but sampled at the MPD communicating with dilated branch ducts that harbored the neoplasm [25]. However, this study, which is the first to compare the efficacy of SPACE among different indications, demonstrated low sensitivity and accuracy in the IPMN group. Therefore, SPACE had limited efficacy in the IPMN group.

The major complication associated with ERCP is PEP, the incidence of which varies widely from 1 to 8% [5]. Past reports have demonstrated that pancreatitis is associated with endoscopic retrograde pancreatography and subsequent pancreatic juice collection or brush cytology (ranging from 0 to 21%) [21,29,30,31]. However, the risk factors for PEP in patients who underwent SPACE are unknown. Mikata et al. reported that PEP occurred in two of the fifty-six conventional group patients (3.6%) and in three of the forty NPD-malignant group patients (7.5%) with suspected pancreatic cancer; however, there were no significant differences in the incidence of pancreatitis between the conventional and SPACE groups [5]. In contrast, our study revealed that the ratio of PEP cases was 7.3% in patients with pancreatic masses and 4.5% in patients suspected of pancreatic cancer without pancreatic masses, all of which were resolved with conservative treatment. Nevertheless, no severe PEP was observed after NPD placement. Kawaguchi et al. reported that NPD placement helps prevent PEP exacerbation [32]. NPD placement, similar to a pancreatic duct stent, has a preventive effect on acute pancreatitis in patients with suspected pancreatic cancer. In contrast, the frequency of PEP was higher in the IPMN group. This study demonstrated that the PEP frequency was high in patients with IPMN (13%), and the SPACE diagnosis of IPMN was an independent risk factor for PEP. Yamakawa et al. reported that PEP occurred in 38.6% of the 44 patients with IPMN who underwent NPD placement, and NPD tube placement was a risk factor for PEP in patients with IPMN [13]. The mucus produced by IPMN may impede the flow of pancreatic juice via the ampulla and NPD tube and induce PEP. Based on the results regarding efficacy and safety, SPACE for IPMN may be ineligible, and patients with suspicions of pancreatic cancer may be a good indication.

The diagnostic ability of EUS-FNA is excellent (sensitivity and specificity were both 90%) and is usually not associated with serious AEs [9,10,11]. However, recent prospective studies have reported that, in 5–8% of patients, EUS-FNA was indicated but was not performed because the mass was too hard to insert the needle; either blood vessels were present in the pathway of the needle, or the mass was too small [33,34]. EUS-FNA cannot be performed in cases of in situ carcinoma and can also be difficult when the tumor is unclear. Furthermore, a meta-analysis reported that the diagnostic yield of EUS-FNA for solid pancreatic lesions (SPLs) <20 mm was inferior to that for SPLs >20 mm [12]. In contrast, our data showed that the diagnostic accuracy was higher in small or early PC than in advanced PC. Previous studies also reported that the sensitivity of pancreatic juice cytology for small pancreatic tumors was higher than that for large tumors [5,7,21]. Furthermore, Nakaizumi et al. suggested that the development of fibrosis at the boundary of the tumor, obstruction of the MPD, or reduced pancreatic exocrine function could prevent cancer cells from flowing into the main pancreatic duct [21]. Although EUS-FNA is the first tool recommended in the Japanese guidelines for obtaining pathological evidence, SPACE was considered when EUS-FNA was difficult or when a small/early pancreatic cancer, including carcinoma in situ, was suspected.

The limitations of our study must be acknowledged when interpreting our results. This study had a retrospective, single-center design. Thus, the optimal indications for SPACE remain unclear. However, the sample size of this study was relatively large regarding the SPACE analysis. Several studies have reported the diagnostic yield and safety of SPACE. However, they included other cytology procedures (simple, brush, and washing cytology). The strength of our study is that it includes SPACE only, which reduces the bias of the safety analysis.

## 5. Conclusions

In conclusion, SPACE is useful and safe in patients with suspected PC, especially those with early-stage PC. However, in patients with IPMN, SPACE has limited efficacy, and the risk of PEP might be high.

## Figures and Tables

**Table 1:** Baseline patient characteristics of the PC and IPMN groups.

	Group A (*n* = 41)	Group B (*n* = 66)	Group C (*n* = 119)	*p*-Value
Median age, year (range)	72 (42–83)	71.5 (33–84)	73 (49–87)	0.16
Sex, male, *n* (%)	28 (68)	38 (58)	66 (55)	0.36
Location of the target lesion, head, *n* (%)	22 (54)	17 (26)	71 (60)	<0.01
Maximum diameter of the MPD, mm (range)	4 (1–13)	4 (2–15)	5 (2–16)	<0.01
History of acute pancreatitis, *n* (%)	6 (15)	21 (32)	5 (4)	<0.01
Size of pancreatic mass, mm (range)	17 (7–55)	–	–	–
Macroscopic type of IPMN, MD/BD/MX, *n* (%)	–	–	12 (10)/62 (52)/45 (38)	–
Risk classification, according to ICG 2017, HRS/WF/others, *n* (%)	–	–	33 (28)/70 (59)/16 (13)	–
The size of NPD tube, 5Fr/6Fr, *n* (%)	41(100)/0(0)	65(98)/1(2)	106(89)/13(11)	<0.01
The median number of cytology samples, times (range)	6 (4–7)	6 (3–7)	6 (1–7)	0.23
Final diagnosis, malignant, *n* (%)	29 (71)	14 (21)	22 (18)	<0.01

Group A, patients with pancreatic mass; Group B, patients without obvious pancreatic mass, 3.2. Group C, IPMN patients; IPMN, intraductal papillary mucinous neoplasms; MD, main duct IPMN; BD, branch duct IPMN; MX, mixed type IPMN; ICG 2017, International consensus Fukuoka guideline 2017; HRS, high-risk stigmata; WF, worrisome feature; NPD, naso-pancreatic drainage.

**Table 2:** Diagnostic ability of SPACE for pancreatic carcinoma and malignant IPMN.

Group (*n*)	Result of SPACE	Malignant		Sp	Se	PPV	NPV	Accuracy
		Yes	No	[95% CI]	[95% CI]	[95% CI]	[95% CI]	[95% CI]
		(*n*)	(*n*)	(%)	(%)	(%)	(%)	(%)
Group A	Positive	TP = 20	FP = 9	69.0	100	100	57.1	78.0
(*n* = 41)	Negative	FN = 0	TN = 12	[49.2–84.7]	[73.5–100]	[83.2–100]	[34.0–78.2]	[62.4–89.4]
Group B	Positive	11	3	78.6	98.1	91.7	94.4	93.9
(*n* = 66)	Negative	1	51	[49.2–95.3]	[89.7–100]	[61.5–99.8]	[84.6–98.8]	[85.2–98.3]
Group C	Positive	6	16	27.3	86.6	31.6	84.0	75.6
(*n* = 119)	Negative	13	84	[10.7–50.2]	[78.2–92.7]	[12.6–56.6]	[75.3–90.6]	[66.9–83.0]
*p*-value for comparison between groups				*p* = 0.002	*p* = 0.039	*p* < 0.001	*p* < 0.001	*p* = 0.004

Group A, patients with pancreatic mass; Group B, patients without obvious pancreatic mass; Group C, IPMN patients; SPACE, serial pancreatic-juice aspiration cytologic examination; IPMN, intraductal papillary mucinous neoplasm; CI, confidence interval; FN, false negative; FP, false positive; NPV, negative predictive value; PPV, positive predictive value; TN, true negative; TP, true positive; Se, sensitivity; Sp, specificity. Sp = TN/(FP + TN), Se = TP/(TP + FN), PPV = TP/(TP + FP), NPV = TN/(TN + FN), accuracy = (TP + TN)/(TP + FP + FN + TN). Fisher’s exact test was performed for comparison between groups.

**Table 3:** The association between pancreatic mass size and the accuracy of SPACE results of Fisher’s exact test for trends showing the negative correlation between pancreatic mass size and the accuracy of SPACE.

Size of Detected Mass	Correct Diagnosis	Incorrect Diagnosis	*p*
1–10 mm	6 (18.8%)	0 (0%)	<0.001
11–20 mm	22 (68.8%)	3 (33.3%)	
≧21 mm	4 (12.5%)	6 (66.7%)	

SPACE, serial pancreatic-juice aspiration cytologic examination.

**Table 4:** Adverse events of SPACE.

	Group A (*n* = 41)	Group B (*n* = 66)	Group C (*n* = 119)	*p*-Value
NPD-related events, *n* (%)	0 (0)	1 (1.5)	5 (4.2)	0.22
Dislocation, *n* (%)	0 (0)	1 (1.5)	4 (3.4)	-
Occlusion, *n* (%)	0 (0)	0 (0)	1 (0.8)	-
Accidental removal, *n* (%)	0 (0)	0 (0)	0 (0)	-
ERCP-related adverse event, *n* (%)				
PEP	3 (7.3)	3 (4.5)	15 (13)	0.20
Mild, *n* (%)	3 (7.3)	2 (3.0)	14 (12)	-
Moderate, *n* (%)	0 (0)	0 (0)	1 (0.8)	-
Severe, *n* (%)	0 (0)	0 (0)	0 (0)	-
Cholangitis, mild, *n* (%)	0 (0)	1 (1.5)	1 (0.8)	1.00

Group A, patients with pancreatic mass; Group B, patients without obvious pancreatic mass; Group C, IPMN patients; IPMN, intraductal papillary mucinous neoplasm; NPD, naso-pancreatic drainage; ERCP, endoscopic retrograde cholangiopancreatography; PEP, post ERCP pancreatitis.

**Table 5:** Risk factors of PEP in patients who underwent SPACE.

Factors		Univariate		
		**OR**	**95% CI**	***p***
Age	>75	0.77	0.27–2.11	0.65
	≤74	1		
Sex	Male	0.76	0.28–2.10	0.64
	Female	1		
MPD size	≥5 mm	0.58	0.18–1.66	0.35
	<5 mm	1		
Indication	IPMN	2.41	0.85–7.92	0.11
	not IPMN	1		
History of	Yes	1.01	0.18–3.80	1.00
acute pancreatitis	None	1		
Location of target	Pbt	1.61	0.59–4.67	0.36
lesion	Ph	1		
Size of NPD tube	6Fr	0.74	0.02–5.44	1.00
	5Fr	1		

PEP, post-ERCP pancreatitis; OR, odds ratio; CI, confidence interval; MPD, main pancreatic duct; Pbt, pancreatic body and tail; Ph, pancreatic head; NPD, naso-pancreatic drainage.

## Data Availability

The data presented in this study are available on request from the corresponding author. The data are not publicly available due to privacy concerns.
